# Shoes for self-managing chronic hip Pain: the SCHIPP randomized clinical trial protocol

**DOI:** 10.1186/s12891-023-06235-x

**Published:** 2023-02-23

**Authors:** Kade L. Paterson, Kim L. Bennell, Ben R. Metcalf, Sarah E. Jones, Penny K. Campbell, Fiona McManus, Karen E. Lamb, Rana S. Hinman

**Affiliations:** 1 Centre for Health, Exercise and Sports Medicine, Department of Physiotherapy, School of Health Sciences, Faculty of Medicine Dentistry & Health Sciences, The University of Melbourne, Melbourne, Australia; 2 Biostatistics Unit, Centre for Epidemiology and Biostatistics, Melbourne School of Population and Global Health, The University of Melbourne, Melbourne, Australia

**Keywords:** Osteoarthritis, OA, Hip, Footwear, Shoes, Clinical trial, RCT, Biomechanics, Pain

## Abstract

**Background:**

Chronic hip pain is common and disabling and is largely due to osteoarthritis (OA). Self-management is recommended by international OA clinical guidelines yet there are few effective treatment options. Footwear has been suggested as a self-management approach, given that foot motion influences hip forces. Currently, guidelines advocate ‘stable supportive’ shoes for people with OA, however this is based solely on expert opinion given no research has investigated whether these shoes are effective at reducing symptoms in people with OA-related chronic hip pain. Therefore, this randomized controlled trial (RCT) aims to determine if stable supportive footwear reduces hip pain during walking compared to flat flexible footwear in people with chronic hip pain consistent with OA.

**Methods:**

This trial is a 6-month, participant- and assessor-blinded, pragmatic, comparative effectiveness, superiority RCT conducted in Melbourne, Australia. We are recruiting 120 participants aged over 45 years with chronic hip pain consistent with OA from the community. Following baseline assessment, participants are randomized to receive either i) stable supportive shoes or ii) flat flexible shoes. Participants are permitted to choose two different pairs of shoes in their allocated group from a range of options that match prespecified shoe classification criteria. They are advised to wear either pair of study shoes daily for a minimum of 6 hours each day for 6 months. The primary outcome is the 6-month change in average hip pain on walking in the last week. Secondary outcomes include changes in other measures of hip pain, symptoms, function in daily living and sports and recreation, hip-related quality of life, pain at other sites, adverse events, and physical activity. Other measures include co-intervention use, adherence, shoe comfort, descriptive characteristics, footwear characteristics, and objective foot measures.

**Discussion:**

This RCT will determine whether stable supportive shoes reduce hip pain during walking more than flat flexible shoes in people with chronic hip pain. Outcomes will help to inform footwear recommendations in international clinical guidelines for OA-related chronic hip pain, which to date have been based solely on expert opinion because of an absence of RCTs.

**Trial registration:**

Australian New Zealand Clinical Trials Registry reference: ACTRN12621001532897.

## Background

Chronic hip pain is a highly prevalent condition that is largely due to osteoarthritis (OA) [1]. Hip OA affects around 40 million people globally and is a leading cause of years lived with disability [2]. With an ageing and increasingly overweight population, the condition is projected to substantially increase over the next decade, leading to significant societal and health care burdens [3]. Pain and stiffness are hallmark symptoms of hip OA [4], and these contribute to difficulties performing daily activities such as sitting and walking [5], which in turn adversely affect quality of life [6]. These symptoms are also major determinants for invasive and costly end-stage hip joint replacement surgery [7]. Although the aetiology of hip OA is multifactorial, it is widely acknowledged that abnormal joint loading plays a role in the pathogenesis of the condition [8]. As such, interventions targeting hip joint loads are an attractive treatment option.

As hip OA has no cure, advice on self-management of symptoms is advised as core management for all people with the condition [9, 10]. One component of self-management recommended by international OA guidelines is that clinicians provide advice to hip OA patients about footwear [9, 11], given that hip joint loading is influenced by foot motion [12]. However, this advice is based on expert opinion because there are no clinical trials of footwear in people with chronic hip pain to inform guidelines and clinical practice. As a result, leading international OA organizations, including the United Kingdom’s National Institute for Health Care Excellence [9], the American College of Rheumatology [10] and the European League Against Rheumatism [11], have all called for research to determine optimal footwear for patients with OA as a leading research priority. Currently, guidelines advocate ‘stable supportive’ shoes for OA patients [9, 11], yet no research has investigated whether stable supportive shoes are effective at reducing symptoms in people with chronic hip pain.

Despite a lack of clinical trials evaluating the effects of stable supportive footwear on hip OA-associated pain, there is preliminary clinical evidence to suggest shoe styles with cushioning features and motion control properties may be beneficial. In a recent large, assessor- and participant-blinded randomised clinical trial, statistically significant reductions in walking knee pain were found in people with knee OA who wore stable supportive shoes for 6 months compared to those allocated flat flexible shoes [13]. Of note, analysis of secondary outcomes in this trial demonstrated improvements in ipsilateral hip pain with stable supportive compared to flat flexible footwear [13]. This is noteworthy, given the sample of people with knee OA recruited in this study were not selected on the basis of having hip pain, yet still reported improvements in hip symptoms. A subsequent study showed that walking in stable supportive shoes resulted in lower measures of knee joint loading forces compared to flat flexible shoes [14], suggesting a mechanism via which these shoes may improve lower limb joint pain. However, another recent biomechanical study of six patients with instrumented hip implants reported that in vivo hip joint contact forces were lowest when participants walked in flat flexible shoes rather than stable supportive shoes [15]. As such, further research comparing the effects of stable supportive shoes to flat flexible shoes in a sample of people specifically with chronic hip pain is warranted.

This study protocol paper describes an RCT of footwear for people with chronic hip pain consistent with OA. Our primary aim is to evaluate whether stable supportive shoes result in lower hip pain during walking compared to flat flexible shoes, when worn over 6 months. Our primary hypothesis is that there will be significantly greater reductions in hip pain with walking in participants wearing stable supportive shoes, compared to those wearing flat flexible shoes. The secondary aim is to determine if stable supportive shoes will lead to improvements in other clinical symptoms (other measures of hip pain, symptoms, function in daily living and sport and recreation, hip-related quality of life, pain at other sites, global improvement in pain, proportion achieving a clinically-relevant reduction in pain, adverse events and physical activity levels) compared to flat flexible shoes.

## Methods

### Study design

This protocol is described using the Standard Protocol Items: Recommendations for Intervention Trials (SPIRIT) [16]. The Shoes for Chronic HIP Pain (SCHIPP) trial is a participant- and assessor-blinded, pragmatic, randomized, superiority clinical trial comparing stable supportive shoes to flat flexible shoes conducted in Melbourne, Australia. The trial was prospectively registered in November 2021 with the Australian and New Zealand Clinical Trials Registry (ACTRN12621001532897). The primary endpoint is 6 months.

### Sample size calculations

Based on our previous data [13], we have assumed a conservative between-participant standard deviation of 2.7 NRS units and correlation between baseline and 6-month scores of 0.20 for our primary outcome of average hip pain intensity during walking. Adjusting for baseline values, and at a significance level of 0.05, we require 48 people per group to achieve 90% power to detect the between-group MCID of 1.8 (out of 10) NRS units [17] for change in hip pain intensity during walking (baseline minus follow up). Assuming 20% loss to follow up, we will recruit a total of 120 participants (60 per group).

### Participants

Participants with chronic hip pain are recruited from the community using advertisements (print and online), social network media (e.g., Facebook), and our clinical network and volunteer database. To be eligible, participants must meet the National Institute for Health and Care Excellence working definition of peripheral joint OA [9], based on their self-reported hip symptoms. Specifically, participants must be aged ≥45 years, experience hip joint pain with activity, and report no hip joint morning stiffness of more than 30 minutes duration. Participants are also required to have hip pain that: (i) has been present for a minimum of 3 months, (ii) is experienced on most days of the previous month, and (iii) has a severity score of > 4 out of 10 on an 11-point numerical rating scale (NRS; 0 = ‘no pain’ and 10=‘worst pain possible’) during walking in the past week.

We are excluding participants if they: (i) were diagnosed by a health professional as having gluteal tendinopathy, referred hip pain from the lumbar spine, greater trochanteric pain syndrome, or femoro-acetabular impingement syndrome, in the past 12 months; (ii) had hip surgery in the previous 6 months, or if they are planning to have surgery in the subsequent 6 months; (iii) had a hip replacement on the most painful side; (iv) currently use foot orthoses, a brace (on any lower limb joint), a gait aid or have modified/customized shoes; (v) had a hip injection in the previous 3 months or if they are planning to have an injection in the subsequent 6 months; (vi) regularly use footwear styles that would limit their ability to wear the study shoes as required (e.g. thongs, high heels, work boots etc); (vii) have any other musculoskeletal condition anywhere in the body that is worse than their study hip pain; (viii) have an inflammatory or systemic joint disease (e.g. fibromyalgia, gout); (ix) have a neurological condition affecting the spine or either lower limb; (x) cannot understand written or spoken English; and (xi) have a foot size (US) that is not between 7 to 12 for women and 8 to 13 for men. If both hips are eligible, we will use the most symptomatic hip for outcome assessments.

### Procedure

**Fig. 1:**
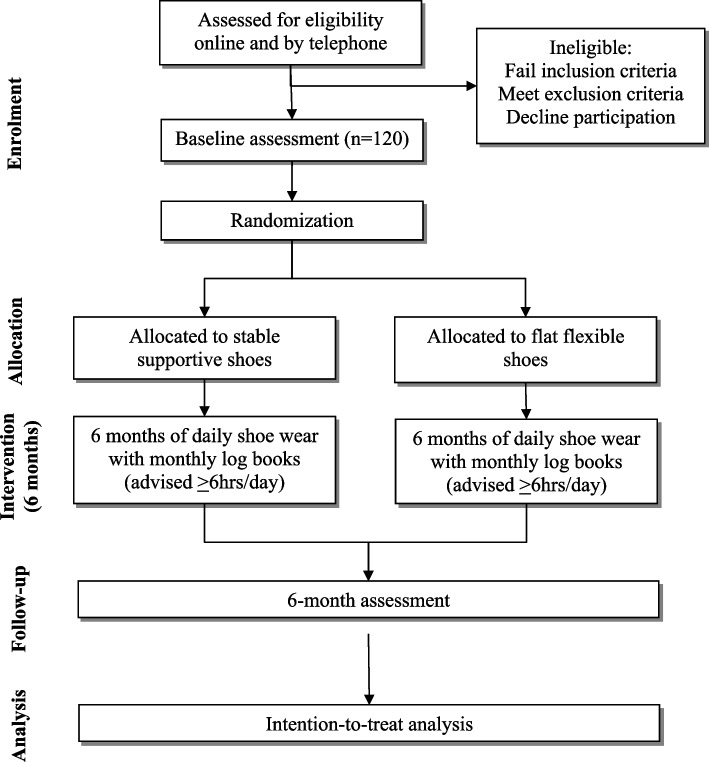
Flow diagram of study phases

The main phases and flow of participants through the trial is outlined in Fig. 1. Volunteers initially complete an online screening form, and if they pass, they are contacted by a study researcher to undergo phone screening. After undergoing screening, participants are provided with information regarding the purpose of the study and are sent the Plain Language Statement and Consent Form. They are then booked for a baseline laboratory visit at the University of Melbourne. Informed consent is obtained from all participants prior to baseline data collection. Approval for all study procedures and documentation has been obtained from the University of Melbourne Responsible Human Research Ethics Committee (2021–22,380–21,619-3).

At the baseline visit, participants complete self-reported outcomes (on paper or electronically) and are then randomized to receive either i) stable supportive shoes or; ii) flat flexible shoes. Participants are advised to wear their allocated study shoes as much as possible when wearing shoes, for a minimum of 6 hours each day, for the subsequent 6 months. We are also collecting participant descriptive measures (e.g., height, weight), objective foot measures (e.g., foot posture and plantar foot pressures) and “usual” footwear characteristics at the baseline visit. At 6 months, participants complete self-reported surveys (paper or electronic) at home to assess primary and secondary outcomes.

### Randomization, blinding and allocation concealment

An independent biostatistician prepared the randomisation schedule using permuted random block sizes. This is saved on REDCap™ using a password and only accessed by a researcher who is not recruiting participants or administering outcome measures. The same study researcher reveals group allocation to participants following completion of baseline primary/secondary outcomes.

We are using limited disclosure to ensure participants are blinded. Participants are informed that we are comparing the effects of two different shoe styles on hip pain, but are not informed which types of footwear we are comparing. We do not inform participants about the shoe features or models in the alternate group. We also do not inform participants regarding the study aims or hypotheses, or about their group allocation, until after the completion of the study. At this time, we provide participants with a plain language summary of the purposes of the study, our aims and hypotheses, and the main study findings. Our trial is also assessor-blinded given our primary and secondary outcome measures are participant-reported, and participants are blinded. Research staff involved with administration of the primary and secondary outcomes are blinded. The statistical analysis plan will be written and published while the biostatisticians are blinded. Main statistical analyses will be performed blinded to group name. The unblinded study researcher responsible for allocating participants to footwear group fits their shoes, measures descriptive characteristics (e.g., height and weight), and assesses objective foot measures and usual shoe characteristics during the laboratory visit.

### Footwear interventions

When planning our previous trial [13], we surveyed people with knee OA (68 women and 43 men) to help select shoes that were acceptable to participants, and ensure that future trial participants would be likely to wear them for at least 6 hours per day for 6 months. The survey presented a number of different shoe styles (for both stable supportive and flat flexible options) from different manufacturers, and in various colours, that could be purchased commercially in Australia. These shoes fulfilled our previously published shoe classification criteria [18] that distinguish stable supportive shoes from flat flexible shoes (Table 1). Although it is possible that other shoe characteristics may influence support (e.g., prescence or absence of laces), shoes matching the characteristics in Table 1 provided preliminary evidence that stable supportive shoes may reduce hip joint pain compared to flat flexible shoes [13]. For the current trial, we selected preferred shoe styles and colour (where available) for the two shoe groups. The chosen shoes for each intervention arm are:

- Stable supportive shoes: ASICS Kayano (men and women), Merrel Jungle Moc (men), Nike Air Max 90 Ultra (women), Rockport Edge Hill (men), and New Balance 624 (women).
- Flat flexible shoes: Merrell Vapor Glove (men and women), Merrell Bare Access (men and women), Vivobarefoot Primus Lite (men and women), Allbirds Tree Skipper (men), Allbirds Tree Lounger (women), Vivobarefoot Mata Canvas (men), and Converse Dainty Low (women).

**Table 1:** Shoe characteristics distinguishing flat flexible shoes compared to stable supportive shoes

	Stable supportive shoes	Flat flexible shoes
**Heel height/thickness**	> 30 mm	< 15 mm
**Shoe pitch**	> 10 mm	< 10 mm
**Arch support/motion control**	Present	Absent
**Sole flexibility**	“Rigid” (Footwear Assessment Tool) [19]	“Minimal” rigidity (Footwear Assessment Tool) [19]
**Weight**^a^	> 300 g	≤200 g

Values obtained from US size 9 shoes

^a^ +/− 10% was permitted for shoe weight

Our previous trial in people with knee OA showed excellent footwear adherence, with participants wearing their shoes for an overall average of 8.4 hours per day across the 6 month trial, and with 81% classified as adherent (wearing their shoes for > 6 hours per day) [13].

While we considered asking participants to wear their own shoes that fulfilled our criteria, our data show most (88%) people wear shoes with mixed features of stable supportive and flat flexible shoes [13]. Thus, people do not typically own shoes that fulfil the specific criteria for each intervention group. In addition, variation in age and wear patterns of participant’s own usual shoes may confound trial findings.

Participants are presented with three different shoe styles from their allocated shoe group and are allowed to choose two different pairs to take home. The shoes are fitted by a study researcher, who advises the participant to wear them for approximately 2 h initially, and then to increase their wear time by an additional 2 h each day. This is to allow for adaptation to the shoes and minimise the potential for an adverse event due to wearing new and potentially unfamiliar shoes. Participants are also advised to wear one pair or alternate both pairs at all times when wearing shoes, for at least 6 hours per day. As shoe models are often discontinued or changed by footwear manufacturers between seasons and years, it is possible that some of our study shoes may become unavailable during the course of the trial. Should this occur, we will source another pair(s) that match the classification criteria listed in Table 1 and will record an amendment to the trial protocol.

### Outcome measures

A summary of key study timepoints including assessments is outlined in Table 2, consistent with SPIRIT recommendations [16].

**Table 2:**
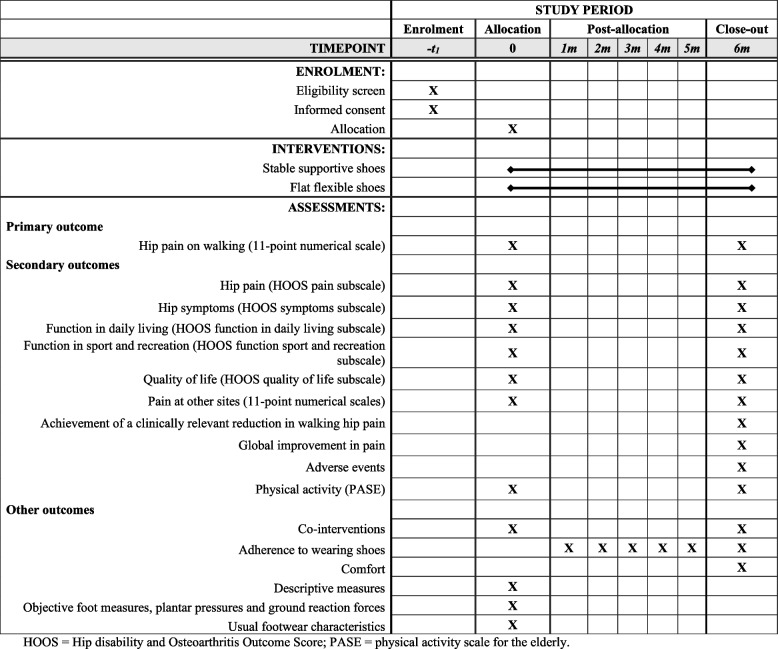
Schedule of enrolment, interventions and assessments.

*HOOS* Hip disability and Osteoarthritis Outcome Score, *PASE* Physical activity scale for the elderly

#### Primary outcome

##### Change in hip pain during walking

Six-month change from baseline in average overall hip pain during walking in the last week, scored on an 11-point NRS, where 0 = no pain and 10 = worst pain possible. This outcome is valid and reliable in OA [20], and is recommended for use in OA clinical trials [21].

#### Secondary outcomes

Secondary outcomes are outlined below. These are measured at baseline and 6 month follow up, unless otherwise specified.

##### Change in the pain subscale of the HOOS

The Hip disability and Osteoarthritis Outcome Score (HOOS) pain subscale contains 10 questions about the severity of hip pain in the last week. Each question has 5 Likert responses that range from ‘None/Never’ (score = 0) to ‘Extreme/Always’ (score = 4) [22]. Scores are transformed to provide a total score ranging between 0 to 100, where lower scores indicate worse pain. The HOOS has been shown to be a valid and reliable outcome measure in hip OA and is responsive to change with treatment [22].

##### Change in the symptoms subscale of the HOOS

The HOOS symptoms subscale contains 5 questions regarding hip symptoms over the previous week [22]. Each question has 5 Likert responses, and scores are transformed to provide an overall score that ranges between 0 to 100.

##### Change in the function in daily living subscale of the HOOS

The HOOS function in daily living subscale is scored using 17 questions regarding hip function over the previous week [22]. Each question has 5 Likert responses, and scores are transformed to give a total score ranging between 0 to 100.

##### Change in the function in sport and recreation subscale of the HOOS

The HOOS function in sport and recreation subscale is scored using 4 questions regarding function during sport and recreational activities over the previous week [22]. Each question has 5 Likert responses, and scores are transformed to provide a total score ranging between 0 to 100.

##### Change in the quality of life subscale of the HOOS

The HOOS quality of life subscale has 4 questions about hip-related quality of life experienced over the previous week [22]. Each question has 5 Likert responses, and scores are transformed to provide a total score ranging between 0 to 100.

##### Change in pain at other sites

Average overall pain during walking in the last week, scored on an 11-point NRS (0 = no pain, 10 = worst pain possible), is assessed for the following sites: (i) contralateral hip, (ii) ipsilateral knee, (iii) contralateral knee, (iv) ipsilateral foot/ankle, (v) contralateral foot/ankle, and (vi) back.

##### Achievement of a clinically-relevant reduction in walking hip pain

Classified based on individual change in hip pain during walking (primary outcome), where any participant achieving a pain reduction greater than or equal to the minimal clinical important difference (MCID) of 1.8 NRS units is classified as achieving a clinically-relevant pain reduction.

##### Achievement of global improvement in pain

Participants rate their overall global change in hip pain at 6 months using a 7-point Likert scale, with terminal descriptors of “much worse” to “much better” in comparison to baseline [23]. Those participants who respond “moderately better” or “much better” are classified as ‘improved’, and all others as ‘not improved’.

##### Adverse events

Adverse events are defined as any problem experienced in the study hip or elsewhere in the body deemed by the participant to be a result of participating in the trial. In addition, the event must have resulted in an increase in pain and/or disability for at least 2 days, and/or necessitated treatment from a health professional, consistent with our previous footwear clinical trials [24–26]. These are participant-reported at 6 months. Participants are also asked to contact the researchers at any time by phone or email to report adverse events. Nature, and number and proportions of participants experiencing adverse events will be reported.

##### Change in physical activity levels

Physical activity is measured using the Physical Activity Scale for the Elderly. This scale contains questions related to occupational, household and leisure physical activities over the previous week [27]. The overall score is between 0 and ‘400 or more’, where a higher score indicates greater physical activity.

##### Comfort

Participants rate their level of comfort with each of their selected allocated shoes at 6 months using an 11-point NRS (where 0 = extremely uncomfortable and 10 = extremely comfortable). Higher scores indicate greater shoe comfort.

#### Other measures

##### Co-interventions

Participants record their previous (past 6 months) use of medications/supplements and other co-interventions (e.g. massage, exercises, injections etc) at baseline and at 6 months follow up. They will be classified as a co-intervention user if they respond that they have used a medication/supplement at least once per week over the past 6 months, or if they report any use of another co-intervention in the last 6 months, at either timepoint, consistent with our previous footwear clinical trials [24–26].

##### Treatment adherence

Adherence to wearing study shoes is assessed at 6 months, unless otherwise indicated, using three methods:

1. Participants rate their level of adherence to the task of wearing their study shoes for > 6 hours/day each day, on average over the previous 6 months, using an 11-point NRS (where 0 = shoes not worn at all and 10 = shoes worn completely as instructed). Higher scores indicate better adherence.
2. Shoe wear is recorded in log-books in the fourth week of the month, as the number of hours each day that each pair of allocated shoes was worn. The hours reported for each pair of shoes will be summed each day to give a daily total and averaged over the 7 days for each month. Number and proportion of adherent (average daily shoe wear ≥6 hours) and non-adherent (average daily shoe wear < 6 hours) participants will be reported for each month, as well as for the entire 6-month intervention period.
3. A single Yes/No question queries if participants stopped wearing their allocated shoes without recommencing wearing them during the study. Those who indicate ‘Yes’ are asked which month they stopped wearing the shoes and to provide a brief description as to why.

##### Descriptive measures

Descriptive measures, recorded at baseline, include height, weight, body mass index (BMI), age, gender, ethnicity, current employment status, level of education, presence/absence of widespread pain (measured using the 2019 revised criteria for chronic widespread pain [28]), symptom duration, comorbidities (assessed using the Self-Administered Comorbidity Questionnaire [29]), and treatment expectation (5-point Likert scale from “no effect at all” to “complete recovery”).

##### Objective foot measures

Objective foot measures, evaluated at baseline, include the Foot Posture Index (FPI [30]), Foot Mobility Magnitude [31], and navicular drop [32]. Foot plantar pressures are recorded during walking using Pedar system insoles (Novel Pedar, Munich, Germany), which are worn inside participant’s own usual shoes and their preferred pair of the two allocated shoes, in random order. Ground reaction forces are also measured while walking: (i) barefoot, (ii) in participant’s own usual shoes and (iii) their preferred pair of the allocated shoes, using two in-ground force platforms. Plantar pressure and ground reaction force data will be reported separately from the main RCT findings as a standalone biomechanics evaluation of the effect of stable supportive compared to flat flexible shoes on these measures.

##### Usual footwear characteristics

Participants bring their most frequently worn pair of shoes (within the past month) into the laboratory at baseline, and these are assessed according to a range of characteristics including shoe weight, heel height, pitch, arch support and flexibility [18]. Number and proportion of participants who currently use flat flexible shoes, stable supportive shoes or shoes of mixed features will be reported.

### Statistical analyses

Intention-to-treat will be used to compare differences between groups for the primary analyses. If more than 5% of data are missing, we will use multiple imputation for the primary analyses and complete-case data for sensitivity analyses. For the primary outcome, differences in mean change in walking hip pain will be compared between groups using a linear regression model, adjusted for baseline values. Continuous secondary outcomes will be analyzed using similar approaches to the primary outcome. We will calculate the proportion of participants that achieve an improvement in NRS hip pain on walking that meets/exceeds the MCID (≥1.8 NRS units [17]) for each group. Risk ratios and risk differences, calculated using logistic regression models, will be used to compare between-group differences in this and other binary outcomes. Poisson regression will be used to compare between-group differences in the number of participants who experience an adverse event. A sensitivity analysis will estimate effects of wearing stable supportive shoes compared to flat flexible shoes assuming full adherence, (> 6 hours per day over 6 months) using an instrumental variables approach [33]. A secondary sensitivity analysis will estimate between-group differences on the primary outcome, adjusted for the presence of chronic widespread pain and baseline values of the primary outcome. Model assumptions will be checked using standard diagnostic plots.

To explore whether the effect of stable supportive shoes on the primary outcome is moderated by either of the pre-specified moderators of baseline BMI or foot posture, interaction terms between randomized group and each potential moderator will be included in regression models for the primary outcome, for each potential effect modifier separately. The rationale for the choice of these moderators is:

- BMI- we hypothesise that decreases in hip pain with stable supportive shoes (relative to flat flexible shoes) will be greater in people with a higher BMI, given that people with an increased body mass have a greater vertical loading rate compared to adults of healthy weight [34], and thus have greater scope for a reduction in loading rate with stable supportive shoes.
- Foot posture- we hypothesise that decreases in hip pain with stable supportive shoes (relative to flat flexible) will be greater in people with a more pronated foot posture, given stable supportive shoes are designed to reduce foot pronation, and foot pronation is associated with lower limb pathology [35].

In addition, flat flexible shoes may be perceived as being less comfortable in people with more pronated feet. Hence, to explore whether the effect of stable supportive shoes on shoe comfort is moderated by foot posture, interaction terms between randomized group and foot posture index score will be included in a linear regression model for the shoe comfort outcome at 6 months. Finally, we are also collecting a range of biomechanical measures that will be used in future studies to investigate potential mechanisms of any improvements in symptoms with footwear.

### Timeline for the SCHIPP study

We gained ethical approval in September 2021. We prospectively registered our RCT on the 10th of November 2021. Participant recruitment began in January 2022 and we expect to recruit our final participants in the first quarter of 2024. Final outcome measures will be completed in the third quarter of 2024.

## Discussion

This protocol has outlined the first RCT investigating the effects of footwear on pain and other symptoms in people with chronic hip pain consistent with osteoarthritis. There have been no clinical trials investigating the efficacy of any footwear for older people with chronic hip pain. However, a previous clinical trial demonstrated statistically significant reductions in hip pain in adults with knee osteoarthritis who wore stable supportive shoes, compared to those wearing flat flexible shoes [25]. This is notable given participants in this previous trial were not required to have hip pain. Subsequent biomechanical research demonstrated that stable supportive shoe reduce knee joint contact forces compared to flat flexible shoes [14], which may also provide a mechanism via which these shoes can reduce hip pain, given increased hip joint forces have been implicated in hip OA pathogenesis [36]. Thus, we hypothesise that 6-month improvements in hip pain with walking will be greater in participants allocated to stable supportive shoes compared to those allocated to flat flexible shoes.

Outcomes from our trial will provide the first RCT evidence regarding the effects of footwear for managing chronic hip pain consistent with OA. Findings of this trial may be used to inform self-management approaches in future OA clinical guidelines to improve the clinical management of people with chronic hip pain.

## Data Availability

The datasets used and/or analysed during the current study will be made available from the corresponding author on reasonable request.

## Abbreviations

BMI: Body mass index
FPI: Foot Posture Index
HOOS: Hip disability and Osteoarthritis Outcome Score
MCID: Minimal clinical important difference
NRS: Numerical rating scale
OA: Osteoarthritis
RCT: Randomized clinical trials
SCHIPP: Shoes for Chronic HIP Pain
SPIRIT: Standard Protocol Items: Recommendations for Intervention Trials
